# Development of a Simple Protocol to Assess Glucose Release After In Vitro Digestion, Allowing Comparison of Starchy Foods

**DOI:** 10.1002/fsn3.70323

**Published:** 2025-05-28

**Authors:** Thomas Montebugnoli, Giorgia Antonelli, Elena Chiarello, Nicholas S. Rivera, Giulia Camporesi, Francesca Danesi, Alessandra Bordoni

**Affiliations:** ^1^ Department of Agricultural and Food Sciences (DISTAL) Alma Mater Studiorum—University of Bologna Cesena Italy; ^2^ Interdepartmental Centre of Agrifood Industry Research Alma Mater Studiorum—University of Bologna Cesena Italy

**Keywords:** glucose release, glycemic index, in vitro digestion, starchy foods

## Abstract

Diets including foods with low glycemic index have been proposed to reverse the rising incidence of obesity and type 2 diabetes. Unfortunately, many common starchy staple foods have a high glycemic index. Several strategies for producing foods with low glycemic index have been proposed, but their application by the food industry is limited by the difficulty and cost of measuring GI in vivo. The aim of the present study was to develop a simple and reliable protocol to assess glucose release after in vitro digestion, and to use it to compare commercial starchy foods. To this purpose, a final starch digestion step was included in the INFOGEST in vitro digestion protocol by adding the enzyme amyloglucosidase, which mimicked the action of brush border enzymes. Glucose release was evaluated in five starchy foods (flour, bread, crackers, and *durum* wheat pasta and gluten‐free pasta), available on the market in two forms produced by the same company, one standard and one rich in fiber (> 6 g/100 g). Glucose release was evaluated 0, 30, 60, and 120 min after the addition of amyloglucosidase. In all foods, glucose release at T0 was very low, confirming that starch digestion by amylases is incomplete. Excluding flours, the lowest and highest amounts of glucose release (28.29 and 49.36 g/50 g of available carbohydrates) were detected in *durum* wheat pasta and gluten‐free pasta, respectively, confirming the high glycemic index previously detected in many gluten‐free products. Notably, when glucose release was expressed as g/50 g of available carbohydrate, the impact of fiber content was negligible. Although the in vitro assessment of glucose release should not be confused with the glycemic index, the herein reported protocol can help predict the impact of foods on glycemia, facilitating the formulation of healthier products.

## Graphical abstract

A simple and reliable protocol has been developed to evaluate glucose release after in vitro digestion and compare some commercial starchy foods available on the market in two forms produced by the same company, one of which is rich in fiber. Excluding flours, the lowest and highest amounts of glucose release were detected in durum wheat pasta and gluten‐free pasta, respectively, confirming the high glycemic index previously detected in many gluten‐free products. Notably, when glucose release was expressed as g/50 g of available carbohydrate, the impact of fiber content was negligible. This protocol can help predict the impact of foods on glycemia, facilitating the formulation of healthier products.

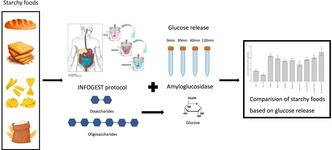

## 1 Introduction

The incidence of obesity and type 2 diabetes (T2D) is increasing worldwide, and nutritional management plays a crucial role in their prevention and treatment (Minari et al. 2024). Diets incorporating foods with low glycemic index (GI) have been proposed as useful means for managing glucose response (Ojo et al. 2018). The GI is the increase in blood glucose concentration in response to a test food consumed under standard conditions and is expressed as a percentage of the area under the curve (AUC) following the consumption of a reference food that the same individual consumes on a different day (Singh et al. 2021). Since the GI value of a food is not based on the characteristics of the individual who consumed it but depends on the characteristics of the food itself (Vega‐López et al. 2018), it serves as a systematic classification system.

Carbohydrates account for 45%–60% of total energy requirements in the human diet (EFSA Panel on Dietetic Products, Nutrition, and Allergies (NDA) 2010), and are among the main determinants of postprandial glycemia. They are directly related to GI, and the form and quantity of carbohydrate‐rich foods in the diet play a fundamental role in the onset of T2D (Feinman et al. 2015). The GI of a food is considered an indicator of health choices and can offer useful guidance on which foods to choose (Zafar et al. 2019). Therefore, obtaining information relating carbohydrate‐rich foods and their GI is crucial. Unfortunately, the GI of many staple foods, such as bread, pasta, and potatoes, is high (Atkinson et al. 2021).

Several strategies for producing foods with low GI have been proposed, mainly focused on slowing the process of starch digestion (Wee and Henry 2020; Yang et al. 2023). In fact, the rate of starch digestion is useful for predicting the glycemic response to starchy foods as it is directly related to the amount of glucose absorbed into the bloodstream. However, the complex relationship between the chemical properties of starch‐rich foods and the digestibility of starch has not yet been fully elucidated. It is known that the digestibility of starch, that is, its conversion into free glucose, is linked to its morphological characteristics, its organization within the crystalline lamellae, the proportion of amylose and amylopectin, and the formation of amylose‐fat inclusion complexes (Bertoft 2017). Non‐starchy components (proteins, fats, and polysaccharide related substances) can interact with starch, changing its structure and making it inaccessible to digestive enzymes (Qadir and Wani 2022), and the processing of starchy foods can affect digestibility by reorganizing the starch structure (Yan et al. 2020). Consequently, the solely chemical composition of a food is not sufficient to predict its GI, and a more direct measurement is needed.

The in vivo determination of GI is very complicated as it involves the supervision of human subjects with intra‐individual variations and is both expensive and cumbersome (Lal et al. 2021). To address these issues, several in vitro digestion methods have been developed to study the digestive behavior of starch‐rich foods (Yang et al. 2023). Despite being simple, convenient, and cost‐effective, these models have rarely been validated with in vivo results. The in vitro static digestion method developed under the COST action INFOGEST has high intra‐ and inter‐laboratory reproducibility, cost‐effectiveness, robustness, allows for easy evaluation of results at each digestion stage (Brodkorb et al. 2019), and it was validated with in vivo results for digestibility of dietary proteins (Sousa et al. 2023). Despite all these advantages, this in vitro digestion method does not consider the last phase of starch hydrolysis. In vivo, disaccharides such as maltose and limit dextrin, which are the products of starch hydrolysis by salivary and pancreatic α‐amylases, are hydrolyzed into glucose by the dual action of two brush border enzymes (BBEs), namely maltase‐glucoamylase and sucrase‐isomaltase (Tannous et al. 2023). BBEs are not included in the INFOGEST protocol (Brodkorb et al. 2019), which only yields a partial release of free glucose from available starch.

The aim of the present study was to develop a simple and reliable protocol to evaluate in vitro glucose release from starchy foods by including in the INFOGEST protocol a final starch digestion step that mimics BBE digestion by adding the enzyme amyloglucosidase (AMG). To verify the reliability of the protocol, glucose release was evaluated in five commercial starchy foods (flour, bread, crackers, and *durum* wheat pasta and gluten‐free pasta). The foods were selected based on their different processing and their availability on the market in two forms produced by the same company, one of which was rich in fiber (over 6 g/100 g, according to the Regulation [EC] 1924, European Commission, 2006). In fact, it has been reported that adding dietary fiber to starchy foods can lower the rate of starch digestion (Qi et al. 2018).

Although other studies have combined the INFOGEST protocol and the addition of AMG to mimic the last phase of starch digestion (Feng et al. 2023; Hammond et al. 2024; Miehle et al. 2024), to our knowledge this is the first one comparing different commercial starchy foods, obtaining results consistent with previous assessment of GI in vivo.

## 2 Materials and Methods

### 2.1 Materials

Unless otherwise specified, chemicals and solvents were of the highest analytical grade and purchased from Merck (Darmstadt, Germany) and Sigma‐Aldrich (St. Louis, MO, USA).

### 2.2 Food Samples

Five different commercial starchy foods were considered: wheat flour, frozen wheat bread, wheat crackers, and *durum* wheat pasta, and gluten‐free corn pasta. Two different types—control and high‐fiber (HF)—of each food, produced by the same company, were studied. All food samples were purchased at a local market.

Wheat flour and crackers were digested without any further processing; frozen bread was defrosted at room temperature, cut into 1 cm slices, and the 5 g to be digested were taken from the central slice, taking care to maintain the same proportion between crust and crumb. The *durum* wheat semolina pasta (fusilli) and the gluten‐free pasta (fusilli) were cooked according to the ISO 7304‐1:2016(E) method, following the cooking time indicated by the manufacturer. After the addition of extra virgin olive oil (1 g of oil/8 g of pasta), the cooked pasta was immediately digested to avoid starch retrogradation.

### 2.3 In Vitro Digestion

Five gram of food were in vitro digested according to the INFOGEST protocol (Minekus et al. 2014) with some modifications. Digestion lasted for 2 min (oral digestion with simulated salivary fluid at pH 7) plus 120 min (gastric digestion with simulated gastric juice containing 2000 U/mL pepsin at pH 3) plus 60 min (duodenal digestion with simulated pancreatic juice containing 10 mM bile and an amount of pancreatin such that the trypsin activity in the final mixture was 100 U/mL at pH 7) at 37°C. At the end of duodenal digestion, samples were centrifuged at 4500×*g* for 10 min at 4°C. The supernatant was aliquoted in 1 mL vials, and 150 μL AMG (230 U/mL in glycerol, final concentration 30 U/mL) or water (T0) were added, as reported by Li et al. (2022). Samples were incubated in a shaking bath at 37°C. Incubation was stopped after 30 (T0 and T30), 60 (T60), or 120 (T120) min by inactivating the enzymes at 100°C for 10 min. Samples were cooled, centrifuged at 10,000×*g* for 5 min at 4°C, and the supernatant was used for glucose determination.

### 2.4 Determination of Amylase Activity

Amylase activity was assessed using the Amylase Activity Assay kit (Sigma‐Aldrich, MO, USA) following the manufacturer's instructions and using ethylidenepNP‐G7 as the substrate. One unit is the amount of the enzyme that cleaves ethylidene‐pNP‐G7 to generate 1.0 mmol of *p*‐nitrophenol per min, which is measured colorimetrically at 405 nm.

### 2.5 Determination of Amyloglucosidase Activity

Amyloglucosidase activity was determined using the Amyloglucosidase Activity Assay kit (Megazyme, Bray, Ireland) following the manufacturer's instructions and using *p‐*nitrophenyl beta‐maltoside plus thermostable beta‐glucosidase as substrate. One unit is the amount of enzyme that releases 1.0 mmol of *p*‐nitrophenol per min from *p‐*nitrophenyl β‐maltoside, which is measured colorimetrically at 400 nm.

### 2.6 Determination of Glucose Content

Glucose concentration was measured with the D‐Glucose Assay Kit using GOPOD reagent (Megazyme, Bray, Ireland) following the manufacturer's instructions. Based on the matrix that was digested, a dilution factor (with water) was chosen for the samples, which were analyzed in duplicate. One hundred microlitesr of water were used as the blank, and 100 μL D‐Glucose solution as standard. Three milliliters of the GOPOD reagent were added to each tube, and after vortexing the tubes were incubated in a hot water bath at 45°C for 20 min. The colorimetric change was measured at 510 nm. Relative to the absorbances of the standard, the content of D‐Glucose was determined using the calculation files downloadable from Megazyme's website (https://www.megazyme.com/d‐glucose‐assay‐kit) and the following equation.

$$D-\text{Glucose}\;\left(\mathrm{\mu g}/0.1\;\mathrm{mL}\right)=\Delta \;\text{Sample}/\Delta \;D-\text{Glucose}$$

### 2.7 Statistical Analysis

Statistical analysis was by the one‐way analysis of variance (ANOVA) with Tukey's as post‐test, and by the Student's *t*‐test, considering *p* < 0.05 as significant.

## 3 Results and Discussion

Several intestinal enzymes are needed for starch digestion (Li et al. 2023): two luminal endo‐glucosidases called alpha‐amylases and different maltases. These maltases are exo‐glucosidases bound to the luminal surface of enterocytes that hydrolyze linear starch oligosaccharides to glucose. In this study, the addition of AMG after 1 h of the duodenal phase of the INFOGEST in vitro digestion facilitated the hydrolysis of terminal α‐1,4 and α‐1,6 D‐glucose residues successively from non‐reducing ends of maltodextrins, thus breaking down available starch completely into glucose. Notably, AMG was added without stopping the pancreatic amylase activity, thus better mimicking the mucosal digestion in vivo.

In the INFOGEST method (Minekus et al. 2014), the amount of pancreatin to be added during digestion is calculated based on the activity of the trypsin enzyme. Since our aim was to evaluate the release of glucose from starch, in preliminary experiments we adjusted the pancreatin amount based on amylase activity (200 U/mL), then compared results obtained in the same sample by including in the duodenal digestion an amount of pancreatin calculated based on the activity of the enzyme trypsin or amylase. Glucose release was evaluated at T0, T30, T60, and T120 of AMG digestion. As shown in Figure S1, no differences were detected at any time point. Based on this, in subsequent experiments the amount of pancreatin to be added during duodenal digestion was calculated according to the INFOGEST protocol.

The nutritional composition of the commercial products considered in the study is shown in Table 1 according to the producer labeling. Ingredients in analyzed foods are reported in Table S1.

**TABLE 1:** Nutritional composition of the commercial products according to the producer label.

Content (g/100 g)	Control	High‐fiber
*Wheat flour*		
Starch	68.7 (75.3 g glucose^a^)	49.5 (54.9 g glucose^a^)
Sugars	1.5	0.5
Fiber	1.6	20
Fats	1.6	1.7
Proteins	12	17
*Frozen bread*		
Starch	45.6 (50.6 g glucose^a^)	38.6 (42.5 g glucose^a^)
Sugars	1.4	0.3
Fiber	4.4	11.3
Fats	1.6	2.5
Proteins	9.7	10
*Crackers*		
Starch	67.7 (74.15 g glucose^a^)	59 (65.5 g glucose^a^)
Sugars	1.3	2
Fibers	2.7	6.1
Fats	15	16
Proteins	9.6	10
*Durum wheat pasta (fusilli)*		
Starch	67.5 (74.3 g glucose^a^)	60.5 (66.6 g glucose^a^)
Sugars	3.5	3.5
Fibers	3	8
Fats	3	2.5
Proteins	13	13
*Gluten‐free pasta (fusilli)*		
Starch	79.4 (88.1 g glucose^a^)	62.1 (68.3 g glucose^a^)
Sugars	0.6	0.7
Fibers	1.7	7.8
Fats	0.8	1.2
Proteins	7.1	12.1

*Note:* Data are expressed as g/100 g.

^a^ Starch weight was converted to glucose weight using 1.11 as the conversion factor (ratio between the weight of anhydrous D‐glucose in starch and the weight of free D‐glucose) (Pérez‐Donado et al. 2023).

**FIGURE 1:**
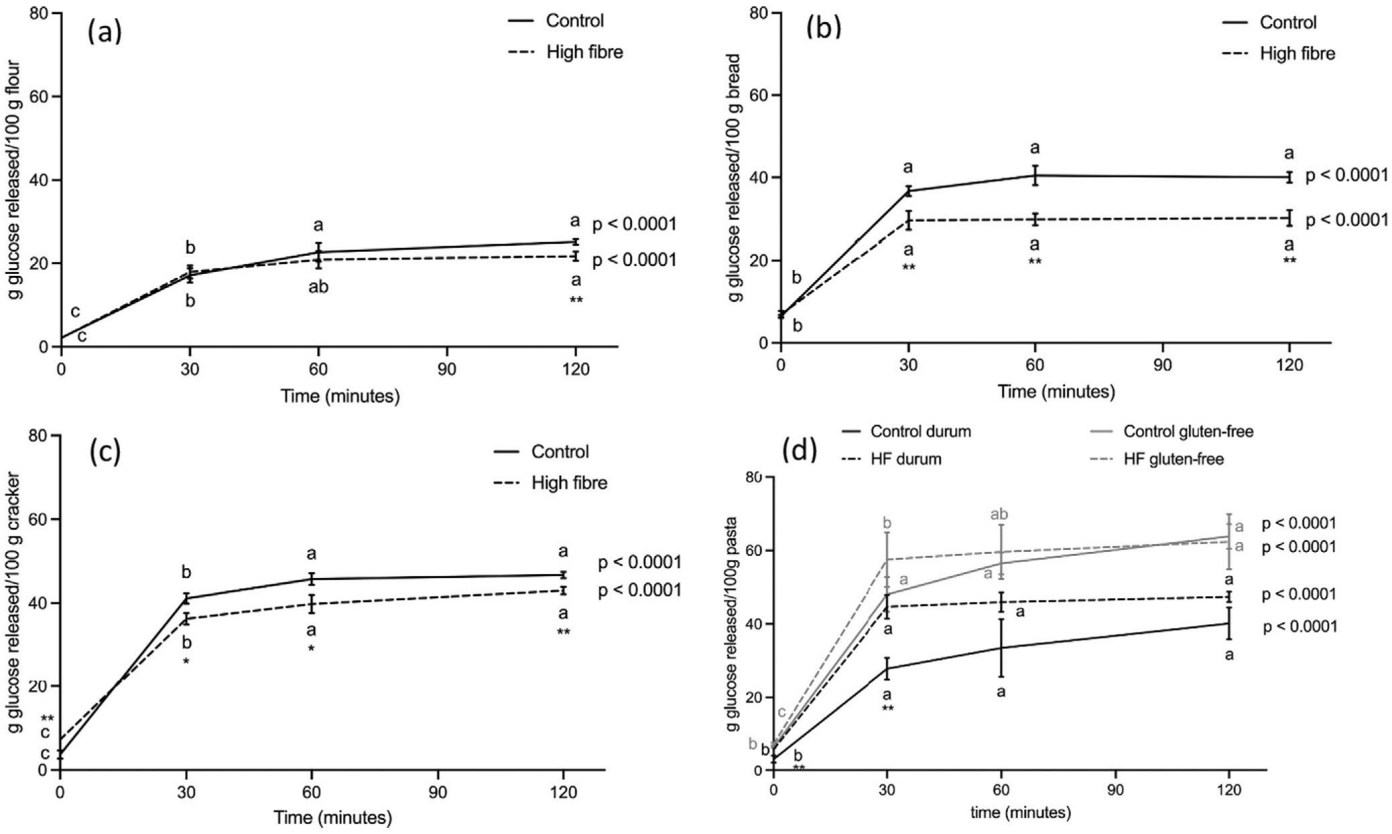
Glucose release after INFOGEST + AMG in vitro digestion of the starchy foods. (a) Flour, (b) bread, (c) crackers, and (d) pasta. Data are expressed as g released glucose/100 g not digested food (raw, in the case of flour and pasta) and are means of three samples coming from different production batches. Statistical analysis was by the Student's *t*‐test comparing the two different samples (control and high‐fiber) at the same time point (*p < 0.05; **p < 0.01) and by the one‐way ANOVA with Tukey's post‐test comparing the same product at the different time points of AMG digestion (different letters indicate statistical significance, at least *p* < 0.05).

Using the described protocol, we evaluated the time course of glucose release during INFOGEST + AMG digestion. In vitro digestion was performed on 5 g of different starchy foods, and results were normalized for 100 g of undigested product (raw, in the case of flour and pasta) (Figure 1).

In flour (Figure 1a), glucose release at T0 was very low and similar in the two products, confirming that starch digestion by amylases is incomplete (Seigner et al. 1987). In both control and HF flour, glucose release increased significantly after 30 min of AMG digestion and continued to increase for the next 30 min, with no significant differences between the two samples. After 2 h, glucose release was significantly greater in control than in HF flour; however, it was lower than in other products. It is conceivable that the low glucose release was linked to the high quantity of non‐gelatinized starch, since starch gelatinization, that is, the heat‐ and moisture‐mediated breakdown of ordered structures in granular starch, is considered a prerequisite for its use. During the starch gelatinization process, also called cooking (Yu et al. 2017), the high temperature and water cause the starch granules to absorb water and swell, softening. This causes the linear and branched chains of starch to be released into the cooking medium and become gelatinized, making starch more susceptible to enzymatic hydrolysis (Gunathilaka and Ekanayake 2015).

At T0, glucose release was low and similar in the two types of bread (Figure 1b); however, it was higher than that of flour (control flour = 2.16 ± 0.16 g/100 g flour; control bread = 6.48 ± 0.32 g/100 g bread), suggesting that starch gelatinization during bread cooking facilitated the release of glucose even during in vitro oro‐gastric‐duodenal digestion. Glucose release significantly increased after the first 30 min of mucosal digestion, without any further significant increase, and was significantly higher in the control compared to HF bread at all time points. It is noteworthy that resistant starch (RS) was included as an ingredient in both HF flour (17%) and HF bread (9.3%). RS, a linear molecule of α‐1,4‐D‐glucan obtained mainly from retrogradation of the amylose fraction (Dery and Lou 2020), cannot be hydrolyzed by digestive enzymes due to its compact molecular structure (Dery and Lou 2020; Wang et al. 2019), and previous reports indicated that the presence of RS has a significant impact on the GI of starchy foods (Afandi et al. 2021).

At T0, glucose release was low in control crackers. HF crackers showed a different kinetic of glucose release; indeed, glucose concentration was higher than in control at T0 and lower at further time points (Figure 1c).

In *durum* wheat pasta, free glucose content was low at the beginning of AMG digestion (Figure 1d). Then, the time course of glucose release was different in the control and HF product. Indeed, it increased at a slower rate in control pasta than in HF pasta, where it plateaued early at T30. At T0, a low and similar release of glucose was also observed in gluten‐free pasta (Figure 1d), and no significant differences were detected at any time of AMG digestion between the control and HF gluten‐free products. When comparing *durum* wheat and gluten‐free pasta, glucose release was greater in the latter, and differences were more evident in control than in the HF variant (control: *p* < 0.001 at any time point; HF: T0 *p* < 0.01; T30 *p* = 0.052; T60 and T120 *p* < 0.05).

**FIGURE 2:**
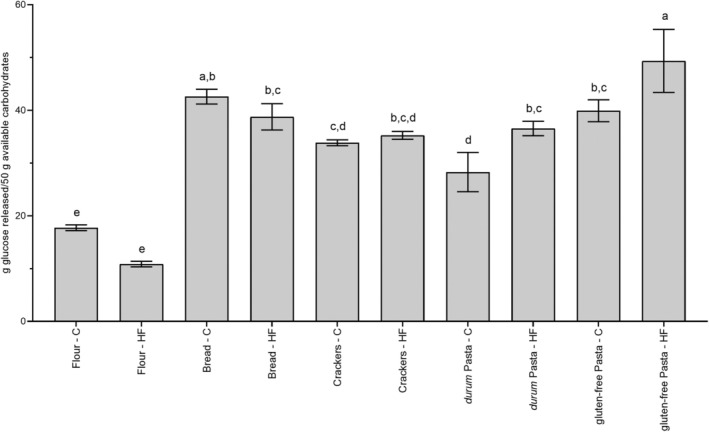
Glucose release after INFOGEST + 120 min AMG in vitro digestion of the starchy foods. Data are expressed as g released glucose/quantity of not digested food (raw, in the case of flour and pasta) containing 50 g available carbohydrates, and are means of three samples coming from different production batches. Statistical analysis was by the one‐way ANOVA with Tukey's post‐test (different letters indicate statistical significance, at least *p* < 0.05).

The described protocol allowed us to compare different starchy foods by evaluating the release of free glucose after in vitro digestion. This should not be confused with the GI. In fact, the GI also takes into account the absorption of glucose by enterocytes, which partly release it into the bloodstream and partly convert it into other metabolites (Chiarello et al. 2022), and the modulation by insulin secretion. However, since much of the information in the literature comes from clinical studies evaluating the GI of starchy foods, which is defined as the incremental area under the blood glucose response curve 2 h after ingestion of a food containing 50 g of available carbohydrates, we renormalized glucose release at T120 on the quantity of foods containing 50 g available carbohydrates to make our results somewhat comparable with those in vivo and to verify their reliability (Figure 2).

Glucose release was confirmed to be very low in flour, where the starch was not gelatinized. Among other food products, the lowest glucose release was observed in control *durum* wheat pasta (28.29 ± 3.73 g/50 g of available carbohydrates). *Durum* wheat pasta has been reported to induce a lower postprandial glycemic response than bread and other wheat products in both healthy subjects and subjects with T2D, probably by virtue of its dense and compact physical structure (dry pasta) and the gluten network that surrounds its starch granules (Di Pede et al. 2021). In gluten‐free products, the absence of gluten affects starch digestibility and raises it, thus increasing the postprandial glycemic response (Scazzina et al. 2015). Accordingly, we observed a higher glucose release in gluten‐free pasta than in *durum* wheat pasta (39.91 ± 2.08 and 49.36 ± 5.96 g/50 g of available carbohydrates in control and HF GF pasta, respectively).

Of note, when glucose release was expressed as g/50 g of available carbohydrate, no significant differences were observed between the control‐HF pairs of flour, bread, and crackers, and in HF pasta (both *durum* and gluten‐free) glucose release was higher than in the corresponding control. As it is reported that dietary fibers serve as a physical barrier in digestion, slowing down the interaction between enzymes and substrates (Qi et al. 2018), our results appear in contrast with the GI‐lowering effect of fiber inclusion in foods. The term “fiber” encompasses a wide range of different compounds, usually classified as insoluble and soluble (viscous) fibers. Although they are generically listed on food labels as “fiber,” their effect on GI is different (Jenkins et al. 1988). We were not aware of the type of fiber present in the foods tested, other than the presence of RS in flour and bread. The positive effect of RS on glucose homeostasis has been confirmed by the EFSA (EFSA Panel on Dietetic Products, Nutrition and Allergies (NDA) 2011) approved health claim “replacing digestible starch with RS induces a lower rise in blood glucose after a meal”. However, there are five types of RS, RS1–RS5, and all evidence for EFSA health claims is based on studies evaluating type 2 RS ingredients derived from high‐amylose maize starch (Walsh et al. 2022). To our knowledge, the effects of other types of RS from different plant origins have not yet been evaluated. The type of fiber present in the formulation of the tested foods could explain the lack of any effect on glucose release and further underscore the imperative need to avoid generalizations and carefully consider the type of fiber when reformulating a food to have a lower glycemic impact. Furthermore, it is noteworthy that the mechanism of action of viscous fiber is related to its ability to reduce the rate of diffusion of nutrients from the lumen of the small intestine, thus reducing their absorption. This may have an impact on the GI of the food, but not on the in vitro assessment of glucose release.

In addition to these possible reasons for the poor effect of the presence of fiber, it should be considered that several studies have not highlighted any changes in postprandial glycemic and insulin responses after the elimination of cereal fiber from foods through refining (Wolever and Jenkins 2001). In particular, in vivo data reported ambiguous results for spaghetti, with some studies showing the same GI regardless of fiber content (Jenkins et al. 1988). It could be argued that the pasta format can modulate the GI and therefore this comparison is difficult, but it is worth noting that Pugnaloni et al. (2022) highlighted a higher GI in whole meal than non‐whole meal fusilli, the same format of pasta considered in this study.

However, based on our results, it could be argued that the reduced glucose release observed in some HF products when the evaluation was done on 100 g of food was mainly related to the replacement of available carbohydrates with fiber rather than a direct effect of fiber on starch hydrolysis. In fact, the effect disappeared when the results are normalized to 50 g of available carbohydrates. Of course, the final effect on glucose release could also have been related to components other than fiber in the tested foods. In a review on the beneficial effects of pulse consumption on blood glucose and insulin levels, it was reported that the positive effect of lentil consumption is probably due to their complex macronutrient content, and both protein and dietary fiber content were identified as potential factors in their lower glycemic response (Clarke et al. 2022). However, direct evidence for both remains inconclusive. In agreement, although in our work 3 out of 5 foods were isoproteic in the HF‐control pair, in two of them (bread and crackers) the HF version showed a lower glucose release (expressed as g/100 g of food), while pasta showed the opposite trend. When glucose release was normalized on 50 g of available carbohydrates, no difference was detected between the HF‐control pairs.

The glucose release from foods can be affected by several factors, including the type and proportion of ingredients, processing technology, and cooking parameters, which act in concert (Cui et al. 2024) Evaluating and explaining which of these characteristics have a greater influence on glucose release was not the aim of our work, but results obtained confirm that it cannot be predicted without a direct measurement.

## 4 Conclusions

The evaluation of glucose release from starch via the in vitro protocol described in this study was easy to perform, cost effective, and had no ethical issues. Using this new protocol, we were able to compare the extent and time course of starch hydrolysis in different commercial foods. Since sucrase and lactase enzymes were not included in the protocol, the foods considered in this study had no or very low sucrose/lactose content.

While not equivalent to GI evaluation, this in vitro protocol can be useful for classifying starchy foods and, once further validated in vivo, it may provide information that will allow consumers to appropriately adapt their food choices. Additionally, since there are many factors that influence GI, including the type of starch, amount and type of fiber, non‐starchy ingredients, particle size of the food, pH, and processing, etc., and the formulation of low GI products is challenging for the food industry, the in vitro protocol reported here can help in the preliminary steps of prototype development, proving advantageous in terms of time and costs.

## Author Contributions

**Thomas Montebugnoli:** data curation (equal), investigation (equal), methodology (equal), validation (equal). **Giorgia Antonelli:** data curation (equal), formal analysis (equal), investigation (equal), methodology (equal), validation (equal), writing – review and editing (equal). **Elena Chiarello:** conceptualization (equal), investigation (equal), methodology (equal), validation (equal). **Nicholas S. Rivera:** data curation (equal), investigation (equal), validation (equal). **Giulia Camporesi:** investigation (equal), validation (equal). **Francesca Danesi:** supervision (supporting), writing – review and editing (equal). **Alessandra Bordoni:** conceptualization (lead), data curation (equal), methodology (equal), resources (lead), supervision (lead), writing – original draft (lead).

## Conflicts of Interest

The authors declare no conflicts of interest.

## Supporting information


**Figure S1.** Glucose release after complete in vitro digestion carried out by calibratingthe addition of pancreatin on the activity of trypsin or amylase.

Table S1.

## Data Availability

The original data presented in the study are openly available in Zenodo at doi: 10.5281/zenodo.14217588.
