# Supplementary figures and images for: Development of a Simple Protocol to Assess Glucose Release After In Vitro Digestion, Allowing Comparison of Starchy Foods
Source: Food Sci Nutr. 2025 May 28;13(6):e70323. doi: 10.1002/fsn3.70323 (PMC12121517; doi:10.1002/fsn3.70323)

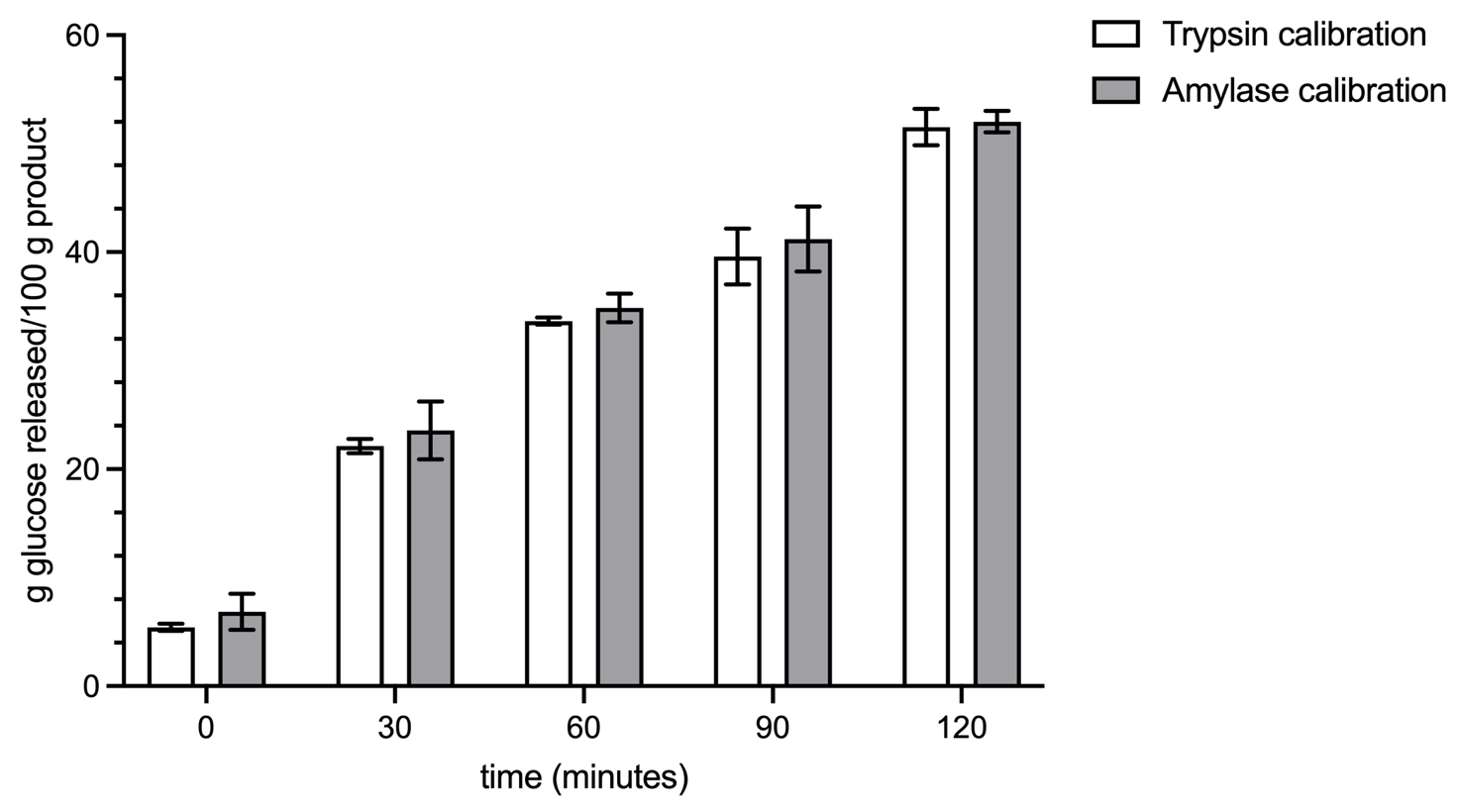

Supplement: Supplementary file 1 — Figure S1. Glucose release after complete in vitro digestion carried out by calibratingthe addition of pancreatin on the activity of trypsin or amylase. [file FSN3-13-e70323-s001.docx]
