# Supplementary material for: Development of a Simple Protocol to Assess Glucose Release After In Vitro Digestion, Allowing Comparison of Starchy Foods
Source: Food Sci Nutr. 2025 May 28;13(6):e70323. doi: 10.1002/fsn3.70323 (PMC12121517; doi:10.1002/fsn3.70323)
Supplement: Supplementary file 2 — Table S1. [file FSN3-13-e70323-s002.pdf]

Table S1. Ingredients in the products analyzed as reported on the manufacturer's label.

**Product**	**Ingredients**
Flour	Type “00” wheat flour
HF flour	Type 1 wheat flour (71.7%), resistant corn starch** (17%), wheat gluten, enzymes
Bread	Type “0” wheat flour (55%), water, sourdough starter (wheat flour, water) (8%), sea salt, brewer’s yeast, malted wheat and barley
HF bread	Stone-ground type “1” wheat flour (39%), water, resistant starch (9.3%), wheat gluten, extra virgin olive oil, brewer’s yeast, sea salt, inulin
Crackers	Wheat flour, vegetable oil (palm and coconut), iodized table salt (1.6%), malted barley flour, salt, yeast (wheat)
HF crackers	Wheat flour, vegetable oil (palm and coconut), wheat bran, Iodized table salt, soluble fiber from wheat starch, malted barley flour, salt, yeast (wheat)
Durum wheat pasta	Durum wheat flour, water
HF durum wheat pasta	Durum whole wheat flour, water
Gluten free pasta	Yellow corn flour (70%), white corn flour (30%)
HF gluten free pasta	Yellow corn flour, resistant starch (9%), soy protein isolate, white corn flour, husked rice flour, bamboo fiber, inulin, emulsifier: vegetable mono- and diglycerides of fatty acids
